# The impact of social deprivation on mortality following hip fracture in England and Wales: a record linkage study

**DOI:** 10.1007/s00198-016-3608-5

**Published:** 2016-04-20

**Authors:** K. Thorne, A. Johansen, A. Akbari, J. G. Williams, S. E. Roberts

**Affiliations:** 1 Swansea University Medical School, Swansea University, Singleton Park, Swansea, SA2 8PP UK; 2 Trauma Unit, University Hospital of Wales, Heath Park, Cardiff, CF14 4XW UK

**Keywords:** Hip fracture, Mortality, Risk factors, Social deprivation

## Abstract

**Summary:**

We used routine hospital data to investigate whether socially deprived patients had an increased risk of dying following hip fracture compared with affluent patients. We found that the most deprived patients had a significantly increased risk of dying at 30, 90 and 365 days compared with the most affluent patients.

**Introduction:**

To identify whether social deprivation has any effect on mortality risk after emergency admission with hip fracture and to determine whether any increased mortality observed among deprived groups was associated with patient and hospital-related factors.

**Methods:**

We used routine, linked hospital inpatient and mortality data for emergency admissions with a hip fracture in both England and Wales between 2004 and 2011. Mortality rates at 30, 90 and 365 days were reported. Logistic regression was used to identify any significant increases in mortality with higher levels of social deprivation and the influence of other risk factors on any increased mortality among the most deprived group.

**Results:**

Mortality rates at 30, 90 and 365 days were 9.3, 17.4 and 29.0 % in England and 8.3, 16.1 and 27.9 % in Wales. Social deprivation was significantly associated with increased mortality in the most deprived quintile compared with the least deprived quintile at 30, 90 and 365 days in England (OR = 1.187, 1.185 and 1.154, respectively) and at 90 and 365 days in Wales (1.135 and 1.203). There was a little interaction between deprivation and other risk factors influencing 30- and 365-day mortality except for patient age, pre-fracture residence and hospital size.

**Conclusions:**

We demonstrated a positive association between social deprivation and increased mortality at 30 days post-admission for hip fracture in both England and Wales that was still evident at 90 and 365 days. We found little influence of other factors on social inequalities in mortality risk at 30 and 365 days post-admission.

## Introduction

There were approximately 75,000 hip fractures in the UK in 2012 and this figure is expected to increase in proportion to the number of elderly individuals in the population [1]. Many older people recovering from a hip fracture have coexisting medical, orthopaedic, psychological or social problems that can make operation and rehabilitation a challenge [2]. Approximately one third of patients will die within 1 year of their hip fracture [2, 3], with mortality rates highest in males [4–8] and patients aged over 80 years [7, 8]. Most deaths are due to pre-existing illnesses rather than the fracture itself, reflecting the impact of comorbidities on mortality rates. It is well known that deprived patients tend to have multiple comorbidities [9]. Some research suggests that mortality rates are significantly higher for deprived patients when compared with more affluent patients following admission for hip fracture [6, 10–12] but there is also evidence of no association [13, 14].

Studies have reported advancing age, male gender [6, 10], delays to surgery and comorbidities [10] as independent predictors of mortality in deprived patients but, to date, few studies have examined the impact of socioeconomic inequalities, and those that have do not provide a consensus. Additionally, there has been little published research on the impact of time on surgery, patient residence prior to their fracture, timing of admission or hospital size. We hypothesise that these factors may contribute to an increase in 30- and 365-day mortality rates in the most deprived quintile compared to the least deprived as a consequence of the poor pre-existing physical status, living conditions and access to services for the majority of people residing in deprived areas.

With no clear consensus available, we investigated associations between social deprivation and mortality following hip fracture in two comparable populations in the UK, England (population 53 million) and Wales (3 million), using the smaller Welsh population to compare the standard error effects across two similar countries with independently collected data sources. Our first objective was to determine whether there was any increased mortality at 30, 90 and 365 days following admission according to increasing social deprivation. Secondly, we determined whether any increased mortality for deprived groups may be affected by factors such as patient age and gender, timing of admission, time to surgery, the presence of dementia, patient’s pre-fracture residence and hospital size.

## Methods

### Study design

We used systematic record linkage of national inpatient and mortality data across England and Wales. All records were accessed through the Secure Anonymised Information Linkage (SAIL) databank, which holds records of inpatient admissions in England (Hospital Episode Statistics—HES) and Wales (Patient Episode Database for Wales—PEDW). All records were linked using a unique anonymised linking field (ALF) in Wales and encrypted Hospital Episode Statistic Identifier (HESID) in England that had been attached to the records of each patient using the patient’s National Health Service (NHS) number or other fields such as date of birth, gender or postcode by applying a probabilistic matching algorithm. More details on the SAIL databank and the MACRAL methodology can be found elsewhere [15, 16].

To identify all deaths that occurred following discharge from hospital as well as in hospital, inpatient data were systematically linked to death certificate data from the Office for National Statistics (ONS). For Wales, we also used the Welsh Demographic Service (formerly known as the Welsh Administrative Register) which also registers deaths for confirmatory purposes.

### Inclusion and exclusion criteria

We selected all emergency admissions to English and Welsh hospitals where hip fracture was recorded as the principal diagnosis on the discharge record. The International Classification of Diseases 10th revision (ICD-10) codes used for hip fracture were S72.0 (fracture of neck of femur), S72.1 (pertrochanteric fracture) and S72.2 (subtrochanteric fracture). We also included S72.9 (fracture of femur, part unspecified) for patients aged 66+ years on admission, but excluded these fractures of unspecified parts of the femur in people aged under 66 as most would refer to fractures of the shaft (e.g. through sporting and traffic injuries) rather than the neck of the femur.

We included patients aged 18 years or over, admitted between January 1, 2004 and December 31, 2011 and followed them up for 12 months to December 31, 2012. Admissions were excluded if they were not emergencies (e.g. elective) or if they occurred within 365 days of a previous hip fracture admission’s discharge date.

### Mortality

Mortality rates at 365 days following the admission were used as the primary outcome measure to determine the short-term impact of social deprivation following hip fracture, with mortality at 30 and 90 days as secondary outcome measures. We included deaths from all causes occurring during the inpatient stay and following discharge.

### Social deprivation

To measure deprivation, we used the Indices of Multiple Deprivation (IMD) 2007 [17] for England and the Welsh Index of Multiple Deprivation (WIMD) 2008 for Wales [18], both of which have been explicitly designed for assigning area-based levels of deprivation to allow socioeconomic evaluations of local and national populations and are updated regularly to reflect the current population. IMD 2007 consists of seven separate domains of deprivation: income (22.5 %), employment (22.5 %), health and disability (13.5 %), education skills and training (13.5 %), barriers to housing and services (9.3 %), crime (9.3 %) and living environment (9.3 %) and is based on 32,482 Lower Super Output Areas (LSOAs; average population = 1500 each). The WIMD 2008 also consists of seven separate domains of deprivation: ‘income’ (23.5 % contribution), ‘employment’ (23.5 %), ‘health’ (14 %), ‘education’ (14 %), ‘access to services’ (10 %), ‘housing’ (5 %), ‘physical environment’ (5 %) and ‘community safety’ (5 %) and is based on 1896 LSOAs. Both indexes provide a deprivation score which was ranked and assigned to one of the five deprivation quintiles (I = least deprived and V = most deprived quintile).

### Risk factors

We assessed a number of key risk factors to determine whether they significantly mediated the relationship between social deprivation and mortality at both 30 and 365 days following admission by using logistic regression. We analysed the impact on mortality for each risk factor, stratified by each subgroup within that risk factor, comparing the least and most deprived cases, using the least deprived quintile as the reference group.

#### Patient demographics

The patient’s age on admission was collected for each case. Age was grouped into <65 years, 65 to 74 years, 75 to 84 years and 85+ years. The patient’s gender was also recorded.

#### Timing of admission

We investigated any impact of the day of admission on mortality by assigning weekdays (Monday 00:00 to Friday 23:59), weekends (Saturday 00:00 to Sunday 23:59) and public holidays (eight per year) which were prioritised over weekdays and weekends in this classification. We also investigated the season of admission (winter = Dec to Feb; spring = Mar to May; summer = Jun to Aug; autumn = Sept to Nov) and calendar years of admission (grouped by 2004–2005, 2006–2008 and 2009–2011).

#### Hospital size

Hospital size at the time of admission was collected from the Health and Social Care Information centre (HSCIC) for England and from Statistics for Wales (StatsWales) for Welsh patients and grouped into 100–399 (small hospital), 400–599 (medium) or 600+ beds (large).

#### Time to surgery

Time to surgery was calculated by determining the difference between the admission date and the date of the first hip fracture-related operation, using the following Office of Population Censuses and Surveys Classification of Surgical Operations and Procedures (4th revision) (OPCS-4) codes: W19.1 (primary open reduction of fracture of neck of femur and open fixation using pin and plate), W24.1 (closed reduction of intracapsular fracture of neck of femur and fixation using nail or screw), W37–W39 (total prosthetic replacement), W46–W48 (prosthetic replacement of head of femur), W58 (resurfacing of hip joint) or W93–95 (hybrid prosthetic replacement). Some of these procedures are performed for other indications (e.g. osteoarthritis), but we included them only when they had been performed as part of the emergency hip fracture admission. We grouped the time to surgery into three categories to reflect NICE guidelines [1], namely surgery on the day of admission or the next day, on the third day or after the third day.

#### Dementia

In our analysis, we defined dementia using ICD-10 codes F00–F03, F05.1 and G30, during the index admission or any admission during the previous 5 years.

#### Pre-fracture residence

As a proxy for pre-fracture mobility, we categorised patients according to their pre-fracture residence: whether they had previously been living in their own home, in a nursing/residential care home or were transferred from another hospital.

### Patient comorbidities

When investigating mortality, we also adjusted for the impact of age group, gender and comorbidities. Specifically, we adjusted for any impact of the following 11 major patient comorbidities using ICD-10 codes recorded in any diagnostic position during the admission or within the previous 5 years from inpatient care records where available: ischaemic heart disease (ICD-10 I20–I25) or other cardiovascular diseases (I00–I15, I26–I52), cerebrovascular disease (I60–I69), other circulatory diseases (I70–I99), malignancies (C00–C97), chronic obstructive pulmonary disease (COPD) (J40–J44), asthma (J45–J46), diabetes (E10–E14), dementia (F00–F03, F05.1, G30), liver disease (K70–K77) and renal failure (N17–N19).

### Methods of analysis

The main study outcome measures were percentage mortality rates, the odds of mortality for the most deprived versus the least deprived and impact of risk factors at 30, 90 and 365 days following admission for each condition using logistic regression.

We reported key demographic characteristics for the most and least deprived cases including age, gender, fracture type and comorbidities and tested for statistical significance using independent sample *t* tests and Pearson’s chi-squared tests. Significance was measured at the conventional 5 % level.

Logistic regression was also used to establish how any higher mortality for deprived groups at 30 and 365 days may be correlated with the following key risk factors: patient age and gender, whether the patient had dementia, the day type, season and year group of admission, hospital size, time to surgery and pre-fracture residence. To do this, we compared mortality in the least and most deprived quintiles, using the least deprived quintile as the reference category, for each stratum of each risk factor. The logistic regression mortality odds ratios were presented with 95 % confidence intervals. Significance was measured at the conventional 5 % level. Thirdly, logistic regression was used to test for any interaction effects on mortality between social deprivation and each of the study risk factors. This would highlight whether there were any significant differences between the mortality odds ratios within each risk factor.

All logistic regression analyses were adjusted for age, gender and the 11 patient comorbidities. We also adjusted the model so that patients with no previous inpatient admissions in the last 5 years, meaning no comorbidities were recorded, did not bias the results.

A Bonferroni correction was applied to account for multiple statistical tests. Results were displayed in tables to indicate whether they were significant before and after the correction was applied.

## Results

Between January 2004 and December 2011, there were 455,862 people admitted with hip fracture in England and 29,733 in Wales.

In England, the mean age at admission was 80.7 years ± 11.6 and males accounted for 26.2 % of cases. In Wales, the mean age was 80.4 years ± 11.1 and males accounted for 25.9 % of cases.

Gender was missing for eight cases from England and no cases from Wales. Social deprivation scores could not be calculated for 5447 cases who did not live in England but were admitted to English hospitals, and 966 cases who did not live in Wales but were admitted to Welsh hospitals. These were not included in the analyses. No other data items in the analysis were missing from the dataset.

### Baseline differences between affluent and deprived quintiles

We found significant differences between the most affluent and the least deprived quintiles for mean age at admission, fracture type and many comorbidities and, for England only, gender and hospital length of stay (see Table 1). When compared with the least deprived cases, the most deprived cases were more likely to be male, younger, presenting with trochanteric fractures and, with the exception of malignancies, were more likely to have comorbidities. In England, they also had a longer inpatient stay, though we did not observe this in Wales.

**Table 1:** Demographics of patients in the least and most deprived quintiles for England and Wales

	England			Wales		
	Least deprived	Most deprived	Sig.	Least deprived	Most deprived	Sig.
No. of cases	86,148	85,422		5333	5765	
Mean age in years (SD)	81.5 (10.9)	78.8 (12.7)	*<0.001*	81.5 (10.3)	79.2 (11.6)	*<0.001*
Gender^a^						
Male	25.7 %	28.6 %	*<0.001*	26.0 %	27.5 %	0.079
Female	74.3 %	71.4 %		74.0 %	72.5 %	
30-day mortality rate (crude %)	8.5 %	9.7 %		8.2 %	9.2 %	
90-day mortality rate (crude %)	16.2 %	18.1 %		15.9 %	17.2 %	
365-day mortality rate (crude %)	27.3 %	30.1 %		26.5 %	29.8 %	
Fracture type						
Fracture of neck of femur	73.6 %	71.7 %	*<0.001*	72.8 %	67.8 %	*<0.001*
Pertrochanteric fracture	22.3 %	24.3 %		23.7 %	28.3 %	
Subtrochanteric fracture	3.2 %	3.2 %		2.9 %	3.3 %	
Fracture of femur, part unspecified	0.8 %	0.8 %		0.6 %	0.6 %	
Mean spell duration (SD)	21.6 (22.5)	24.7 (25.4)	*<0.001*	23.9 (33.2)	23.6 (30.6)	0.704
Comorbidities during previous 5 years						
Acute myocardial infarction	5.5 %	6.5 %	*<0.001*	6.8 %	7.1 %	0.525
Cerebrovascular disease	13.6 %	15.7 %	*<0.001*	17.4 %	18.2 %	0.275
Other circulatory disease	18.0 %	21.1 %	*<0.001*	30.9 %	31.8 %	0.316
Malignancies	13.2 %	12.7 %	*0.003*	16.2 %	14.5 %	*0.011*
Liver disease	1.6 %	3.3 %	*<0.001*	1.4 %	2.9 %	*<0.001*
COPD	8.9 %	18.8 %	*<0.001*	13.0 %	21.7 %	*<0.001*
Asthma	8.1 %	11.7 %	*<0.001*	9.9 %	14.8 %	*<0.001*
Diabetes	11.6 %	15.3 %	*<0.001*	13.0 %	16.9 %	*<0.001*
Renal failure	10.8 %	13.5 %	*<0.001*	10.6 %	11.1 %	0.375
Dementia	23.1 %	24.3 %	*<0.001*	22.1 %	23.8 %	*0.042*

Significance was measured at the 5 % level using chi-squared tests or *t* tests

All significant results are set in italic

^a^Gender was missing for eight cases in England

### Mortality, demographics and social deprivation

In England, mortality at 30 days was 9.3 %, at 90 days was 17.4 % and at 365 days was 29.0 % whilst in Wales, the rates were 8.3, 16.1 and 27.9 %, respectively.

Mortality rates at 30 days were highest in the over 85 age group (13.6 % in England and 11.5 % in Wales) and higher in men (12.2 % in England and 11.0 % in Wales) compared with women (8.3 and 7.4 %). After Bonferroni corrections were applied, social deprivation was significantly associated with an increase in mortality at 30 days in England (most deprived = 1.187 compared with least deprived) but the increase was not significant in Wales (1.136). At 90 and 365 days, mortality rates were significantly increased in both populations (1.185 and 1.154 in England, and 1.135 and 1.203 in Wales; see Table 2).

**Table 2:** Thirty-day mortality odds ratios at 30, 90 and 365 days following hip fracture according to age, gender and social deprivation, 2004 to 2012

Risk factor		Adjusted† 30-day OR (95 % CI)	Adjusted† 90-day OR (95 % CI)	Adjusted† 365-day OR (95 % CI)
England				
Gender^a^	Male	Reference	Reference	Reference
	Female	*0.608* (*0.595*, *0.622*)*	*0.641* (*0.629*, *0.653*)*	*0.651* (*0.640*, *0.661*)*
Social deprivation^b^	I (least deprived)	Reference	Reference	Reference
	II	*1.094* (*1.058*, *1.131*)*	*1.075* (*1.048*, *1.104*)*	*1.046* (*1.023*, *1.070*)*
	III	*1.116* (*1.079*, *1.153*)*	*1.097* (*1.069*, *1.126*)*	*1.075* (*1.052*, *1.099*)*
	IV	*1.157* (*1.119*, *1.196*)*	*1.153* (*1.123*, *1.183*)*	*1.122* (*1.097*, *1.147*)*
	V (most deprived)	*1.187* (*1.147*, *1.228*)*	*1.185* (*1.154*, *1.217*)*	*1.154* (*1.128*, *1.181*)*
Wales				
Gender	Male	Reference	Reference	Reference
	Female	*0.641* (*0.583*, *0.705*)*	*0.664* (*0.616*, *0.714*)*	*0.661* (*0.620*, *0.705*)*
Social deprivation^b^	I (least deprived)	Reference	Reference	Reference
	II	0.993 (0.863, 1.142)	0.970 (0.871, 1.081)	1.093 (0.998, 1.198)
	III	0.981 (0.856, 1.123)	1.046 (0.943, 1.160)	*1.132* (*1.037*, *1.237*)
	IV	0.963 (0.838, 1.106)	1.089 (0.981, 1.209)	*1.151* (*1.053*, *1.259*)
	V (most deprived)	1.136 (0.991, 1.302)	*1.135* (*1.022*, *1.261*)	*1.203* (*1.100*, *1.317*)*

Italic font denotes significance at the 5 % level

*Denotes significance after applying a Bonferroni correction for each condition (*p* ≤ 0.00167)

†The OR for gender is adjusted for age group and comorbidities. All other factors were adjusted for age group, gender and comorbidities

^a^Gender was missing for 26 cases in England

^b^Social deprivation scores were missing for 5447 cases in England and 966 cases in Wales

### Effect of factors on the increased 365-day mortality with social deprivation

Tables 3 and 4 report the mortality rates for quintiles I and V, along with the adjusted 30- and 365-day mortality risk associated with each of the factors listed for England and Wales, respectively.

**Table 3:** Mortality risk at 30 and 365 days following admission for hip fracture in England according to deprivation and key risk factors

Risk factor		No. of admissions		30-day mortality rate (%)		Adjusted† 30-day mort OR	(95 % CI)	365-day mortality rate (%)		Adjusted† 365-day mort OR	(95 % CI)
		Least deprived	Most deprived	Least deprived	Most deprived			Least deprived	Most deprived		
Age	<65 years	6259	10,432	1.7	2.5	*1.151*	*0.905*, *1.464*	6.2	9.8	*1.209*	*1.052*, *1.389*
	65–74 years	9803	12,331	3.5	5.4	*1.208*	*1.050*, *1.390*	13.0	19.2	*1.215*	*1.119*, *1.319*
	75–84 years	31,305	31,321	6.4	9.1	*1.269*	*1.193*, *1.350*	22.3	29.0	*1.216*	*1.170*, *1.265*
	85+ years	38,781	31,338	12.5	14.4	*1.138*	*1.088*, *1.190*	38.4	42.2	*1.090*	*1.056*, *1.125*
Gender	Male	22,148	24,443	11.9	11.4	*1.130*	*1.063*, *1.200*	34.0	33.2	1.143	1.094, 1.195
	Female	63,994	60,977	7.3	9.1	*1.217*	*1.166*, *1.269*	25.0	28.9	1.157	1.125, 1.189
Day of the week	Weekdays (Mon–Fri)	61,716	60,829	8.5	9.7	1.179	1.132, 1.228	27.3	30.1	1.150	1.119, 1.182
	Weekends (Sat–Sun)	22,511	22,668	8.3	9.7	1.218	1.139, 1.304	27.2	30.0	1.156	1.105, 1.209
	Public holidays	1921	1925	9.2	10.2	1.204	0.959, 1.511	28.3	30.6	1.210	1.036, 1.414
Season of admission	Winter	22,482	22,547	9.3	10.5	1.161	1.088, 1.239	28.0	30.8	1.141	1.091, 1.193
	Spring	21,482	21,305	8.4	9.5	1.188	1.108, 1.274	27.0	29.8	1.168	1.115, 1.223
	Summer	20,965	20,672	7.8	9.3	1.228	1.142, 1.320	26.9	29.3	1.130	1.077, 1.184
	Autumn	21,219	20,898	8.3	9.6	1.186	1.105, 1.272	27.4	30.5	1.172	1.118, 1.229
Year of admission	2004–2005	19,867	21,361	9.8	10.7	1.125	1.052, 1.203	29.3	31.5	1.116	1.065, 1.169
	2006–2008	31,892	32,022	9.1	10.4	1.185	1.121, 1.252	28.4	31.4	1.169	1.126, 1.214
	2009–2011	34,389	32,039	7.2	8.4	1.188	1.120, 1.261	25.3	27.9	1.107	1.065, 1.150
Hospital size	100–399 beds	13,282	10,657	8.4	9.2	*1.238*	*1.162*, *1.319*	27.4	29.2	*1.077*	*1.012*, *1.147*
	400–599 beds	29,371	22,541	8.3	9.8	*1.135*	*1.070*, *1.205*	26.8	30.4	*1.028*	*1.158*, *1.261*
	600+ beds	25,948	33,793	8.5	9.5	*1.265*	*1.175*, *1.362*	27.8	29.7	*1.087*	*1.044*, *1.131*
Time to surgery	Same day	12,014	10,159	6.1	6.3	1.105	0.985, 1.239	22.7	23.8	1.115	1.040, 1.196
	Next day	28,128	25,792	6.2	7.1	1.191	1.110, 1.278	23.1	25.4	1.122	1.074, 1.171
	Third day	12,019	11,241	7.2	7.8	1.134	1.024, 1.255	25.8	27.5	1.094	1.025, 1.166
	After 3 days	12,948	14,220	8.4	8.9	1.131	1.035, 1.235	33.1	34.6	1.097	1.038, 1.159
Dementia	No	66,214	64,681	7.6	9.1	1.240	1.189, 1.292	22.2	25.4	1.226	1.192, 1.261
	Yes	19,934	20,741	11.3	11.7	NA	NA	44.4	44.6	1.016	0.975, 1.058
Pre-fracture residence	Own home	78,713	79,849	8.4	9.7	*1.201*	*1.159*, *1.245*	27.2	29.8	1.149	1.122, 1.178
	Nursing/residential home	799	1492	16.1	15.1	*0.851*	*0.664*, *1.092*	47.9	49.5	1.020	0.850, 1.224
	Transfer	5922	3517	9.1	8.4	*0.994*	*0.850*, *1.163*	27.9	28.6	1.099	0.991, 1.219

Quintile I was used as the reference group. The ORs of quintile V are reported in the table

NA indicates regression analysis was not possible

Italic font denotes significant interaction effects at the 5 % level

†The OR for age group was adjusted for gender and comorbidities; gender was adjusted for age group and comorbidities; all other factors were adjusted for age group, gender and comorbidities

**Table 4:** Mortality risk at 30 and 365 days following admission for hip fracture in Wales according to deprivation and key risk factors

Risk factor		No. of admissions		30-day mortality rate (%)		Adjusted† 30-day mort OR	(95 % CI)	365-day mortality rate (%)		Adjusted† 365-day mort OR	(95 % CI)
		Least deprived	Most deprived	Least deprived	Most deprived			Least deprived	Most deprived		
Age	<65 years	355	600	0.8	3.0	*3.297*	*0.927*, *11.28*	5.1	9.0	*1.476*	*0.798*, *2.732*
	65–74 years	640	916	3.6	6.7	*1.711*	*1.026*, *2.854*	12.0	18.0	*1.438*	*1.046*, *1.977*
	75–84 years	2020	2143	6.4	9.7	*1.501*	*1.183*, *1.905*	22.5	30.6	*1.421*	*1.223*, *1.652*
	85+ years	2318	2106	12.3	11.7	*0.886*	*0.734*, *1.069*	37.4	39.9	*1.036*	*0.912*, *1.177*
Gender	Male	1386	1584	11.0	11.8	1.227	0.966, 1.559	33.2	33.4	1.199	1.008, 1.427
	Female	3947	4181	7.3	8.3	1.104	0.932, 1.308	24.2	28.4	1.222	1.098, 1.361
Day of the week	Weekdays (Mon–Fri)	3862	4120	7.9	9.7	*1.280*	*1.088*, *1.506*	26.6	30.4	1.227	1.103, 1.365
	Weekends (Sat–Sun)	1349	1516	9.1	8.1	*0.904*	*0.686*, *1.192*	26.1	28.2	1.177	0.979, 1.414
	Public holidays	122	129	12.3	6.2	*0.547*	*0.205*, *1.459*	27.9	27.9	1.253	0.639, 2.457
Season of admission	Winter	1391	1560	9.3	9.7	1.090	0.840, 1.414	28.5	30.4	1.216	1.021, 1.448
	Spring	1326	1440	8.7	9.5	1.116	0.849, 1.466	26.2	29.9	1.201	0.998, 1.445
	Summer	1285	1387	7.8	9.0	1.148	0.862, 1.530	26.1	29.8	1.206	1.003, 1.450
	Autumn	1331	1378	7.4	8.6	1.240	0.925, 1.664	25.2	28.7	1.221	1.011, 1.475
Year of admission	2004–2005	1188	1420	8.7	10.6	1.398	1.059, 1.847	24.7	31.6	1.571	1.297, 1.903
	2006–2008	2076	2158	8.7	8.6	0.982	0.785, 1.229	27.8	30.3	1.158	1.001, 1.339
	2009–2011	2069	2187	7.7	9.0	1.182	0.940, 1.486	26.3	28.0	1.094	0.941, 1.272
Hospital size	100–399 beds	189	264	6.3	9.8	1.316	1.055, 1.642	23.3	29.2	*1.421*	*0.844*, *2.392*
	400–599 beds	1666	2685	8.8	10.5	0.961	0.791, 1.167	28.3	30.5	*1.231*	*1.060*, *1.430*
	600+ beds	3260	2552	8.6	8.5	1.933	0.446, 8.382	26.7	29.3	*1.123*	*0.989*, *1.274*
Time to surgery	Same day	671	717	6.1	7.1	1.184	0.754, 1.857	20.0	24.4	1.364	1.026, 1.813
	Next day	1615	1824	7.1	8.2	1.235	0.947, 1.609	23.4	26.1	1.198	1.010, 1.421
	Third day	665	662	5.7	6.5	1.200	0.746, 1.928	24.2	28.4	1.287	0.980, 1.689
	After 3 days	1009	986	7.3	8.3	1.217	0.861, 1.720	32.0	35.4	1.257	0.992, 1.493
Dementia	No	4153	4395	7.8	8.4	1.114	0.945, 1.313	22.2	24.8	1.234	1.105, 1.378
	Yes	1180	1370	10.1	12.0	1.234	0.955, 1.595	41.9	45.5	1.170	0.993, 1.377
Pre-fracture residence	Own home	4899	5257	7.8	8.8	*1.172*	*1.011*, *1.360*	25.5	28.4	*1.204*	*1.097*, *1.331*
	Nursing/residential home	236	299	20.8	15.7	*0.631*	*0.392*, *1.017*	51.7	51.2	*0.923*	*0.641*, *1.331*
	Transfer	177	186	4.5	9.1	*1.860*	*0.690*, *5.016*	20.9	33.9	*2.062*	*1.209*, *3.517*

Quintile I was used as the reference group. The ORs of quintile V are reported in the table

Italic font denotes significant interaction effects at the 5 % level

†The OR for age group was adjusted for gender and comorbidities; gender was adjusted for age group and comorbidities; all other factors were adjusted for age group, gender and comorbidities

#### Patient demographics

There was a significant interaction with age group at 30 and 365 days in England (*p* < 0.001 for both time points) and Wales (*p* < 0.001 and *p* = 0.008, respectively), with patients aged over 85 in the most deprived quintile showing a significant and lower odds ratio than the other age groups at 30 and 365 days in England (see Table 3) and Wales (see Table 4).

There was also a significant interaction with gender at 30 days in England (*p* = 0.001), with females in the most deprived quintile having a significantly higher mortality risk. There was no significant interaction effect at 365 days, with males and females showing similar mortality risk. In Wales, males had a higher risk at 30 days and females at 365 days, and neither of which showed a significant interaction effect.

#### Timing of admission

There was a significant interaction effect with weekday at 30 days in Wales (*p* = 0.024), with patients in the most deprived quintile (compared with the least deprived quintile) admitted Monday–Friday having a significantly higher mortality risk than those admitted at the weekend or public holidays. However, there was no significant interaction effect at 365 days, nor for England at 30 or 365 days.

#### Hospital size

There was a significant interaction effect with hospital size at 30 and 365 days in England (*p* = 0.029 and *p* < 0.001, respectively) and for 365 days in Wales (*p* = 0.006).

In England, mortality risk for patients from the most deprived quintile was greatest for large (600+ beds) hospitals at both 30 and 365 days. The medium-sized hospitals had the lowest risk of all at 30 and 365 days. In Wales, there was no significant interaction effect at 30 days but at 365 days, the small hospitals (100–399 beds) had a significantly higher mortality risk than the other hospital groups.

#### Time to surgery

Next day, surgery had the highest odds ratio for 30 and 365 days in England and 30 days in Wales, but there was no significant interaction effect between social deprivation and time to surgery at 30 or 365 days for England or Wales.

#### Dementia

In England, there were insufficient numbers to calculate the odds ratio for patients with dementia who died within 30 days. At 365 days, not having dementia appeared to significantly increase the mortality risk for the most deprived compared with the least deprived quintile. In Wales, the same effect was seen at 365 days but at 30 days, neither odds ratio was significant. There were no significant interaction effects noted.

#### Pre-fracture residence

There was a significant interaction effect for 30-day mortality in England and both 30- and 365-day mortality in Wales. In England, patients from the most deprived quintile admitted from their own home had a significantly higher 30-day mortality risk than patients admitted from a nursing or residential home, or who were transferred in. At 365 days, the mortality risk was still the highest in this group but not significantly so. In Wales, there was a significantly higher risk of 30- and 365-day mortality for patients transferred to the hospital.

## Discussion

We found that social deprivation was significantly associated with higher mortality at 30, 90 and 365 days following an emergency admission for hip fracture in England and at 90 and 365 days in Wales. We also found that patient age, hospital size and pre-fracture residence were significantly associated with mortality in those who were from deprived areas in both populations.

Our 30-day mortality rates of 9.3 % in England and 8.3 % in Wales were comparable with those reported by other hip fracture studies in the UK [10, 19–23]. The same was true for our 365-day mortality rates of 29.0 % in England and 27.9 % in Wales [21–25]. The significant association we found between mortality risk and social deprivation has also been reported by other UK [3, 6, 10, 26] and international [6] studies.

Our data showed that deprived patients were younger on admission. The influence of age on mortality after hip fracture has been extensively described [4–8, 10, 21, 27–30], and our study suggests that the increased mortality risk seen in these deprived patients reflects an increased rate of comorbidities compared to the most affluent patients—a trend that has been reported by others [12].

Other key predictors of mortality following hip fracture include male gender [4–8, 10, 21, 27–30], comorbidities [4, 8, 31, 32], dementia [8, 30], osteoporosis [4], fracture severity [8, 21, 27, 31], surgical delays [10, 32] and post-operative complications [32], living in a nursing or residential home [8], poor pre-injury walking capacity [8] and poor social contact [7]. We hypothesised that many of these predictors would be influenced by patients’ social deprivation status and might increase mortality risk for the most deprived patients. Unfortunately, HES and PEDW do not capture facture severity, walking capacity, post-operative complications or social contact, and comorbidities are not rigorously recorded on records in either country. For example, only 10 % of women with a hip fracture had osteoporosis recorded as a comorbidity. Consequently, we were unable to include these factors into any analyses.

Dementia has been reported as playing a major role in increasing mortality risk in patients with hip fracture as patients have a lower probability of functional recovery at discharge and 6 months post-discharge [33]. Our study showed an increased risk of mortality within 30 days, but not at 365 days, suggesting that the impact of dementia is most crucial during the acute admission, surgery and rehabilitation.

Mortality risk was significantly different in England at 30 and 365 days with the largest hospitals showing the higher mortality rates for deprived patients. In Wales, higher mortality rates were seen in the largest hospitals at 30 days, although this was not significantly different to the other hospital groups. However, at 365 days, it was the smaller hospitals whose mortality rates were significantly higher than the other groups.

Time to surgery and complications after surgery contribute to increased mortality rates. Whilst we were able to investigate the impact of surgical delays according to deprivation status, the administrative data lacked sufficient detail to explore post-operative complications, but there is evidence to suggest that low income is associated with a higher risk of acute medical events and infections [13]. Our data showed that the most deprived patients had a higher mortality rate following surgery than the most affluent patients at 30 and 365 days, and that this difference increased with greater delay to surgery. However, there was no significant interaction effect, so we cannot conclude that the mortality risks were significantly different according to time to surgery. A meta-analysis of hip fracture studies reported that operating beyond 48 h may increase the odds of 30-day mortality by 41 % and of 365-day mortality by 32 % [34], but this remains a complex question since many delays to surgery are a consequence of comorbidities that need assessment or treatment before surgery and anaesthesia can go ahead [35]. People from deprived areas are known to be at higher risk of multiple comorbidities than their affluent counterparts [9], but there is also evidence that socioeconomic deprivation is associated with lower rates of early intervention [6, 13]. If so, then the additional delays experienced by deprived patients might result in higher mortality rates for this group.

The patient’s residence pre-injury will affect mortality risks, with people admitted from a nursing or residential care home, experiencing higher mortality [21]. When we examined patients’ pre-admission residence, we found that in England, 30- and 365-day mortality rates for people admitted from home were higher for deprived than for affluent patients, significantly so at 30 days. In Wales, the highest mortality at both 30 and 365 days was seen in deprived patients transferred to the hospital from another healthcare provider, compared with affluent patients.

Major strengths of the study are its size, covering more than 455,000 cases of hip fracture in England and 29,700 in Wales. The methodology was based on systematic, validated record linkage of inpatient and death certificate to identify all admissions and all deaths that occur during the inpatient stay and following discharge from hospital. Finally, using Welsh data allowed us to compare our findings in a similar, albeit smaller population.

As with other large-scale studies that used NHS administrative health data, this study lacked detailed information about patient disease history or any severity indicators. We were also unable to determine the exact time elapsed until surgery was performed as this information is only recorded at date level with no time field available in either HES or PEDW. We did not combine both populations as the measures of deprivation used in each country were based on different domains with no validated method for merging the two.

Whilst the inclusion of alcohol and substance abuse would have been useful additions to our modelling, the quality of that data was extremely poor and could not be used. Additionally, we were unable to use the Charlson Comorbidity Index in accordance with its requirements as the UK regulations prohibit the access of HIV status in routine data. However, the comorbidities used in this study were based on other measures in that index wherever possible.

Social deprivation refers to problems caused by a general lack of resources and opportunities and not just money. The association between social deprivation and increased mortality risk is multifaceted, and a patient’s pre-existing and baseline clinical and psychological status may contribute to any relationship between social deprivation and mortality. Mortality following hip fracture is usually attributed to underlying ill health [1], but poor social contact pre-injury has also been linked with increased mortality risk [7], and other deprivation-related factors including reduced resources, lower education status, poor lifestyle and social contact, reduced likelihood of preventative medication, and poor mental health, particularly dementia, may also play a part. However, when interpreting these findings of an association between deprivation and increased mortality, it is important to remember the ecological fallacy: not everyone living in a deprived area is deprived, and that not all deprived people live in deprived areas.

## Conclusions

We have demonstrated a clear association between social deprivation and increased mortality following emergency admission for hip fracture in the two UK populations. The study findings also suggest that patient age, hospital size and pre-fracture residence are factors that play a part in this association in both English and Welsh populations.
